# The Effect of Pre-Quarantine Physical Activity on Anxiety and Depressive Symptoms during the COVID-19 Lockdown in the Kingdom of Saudi Arabia

**DOI:** 10.3390/ijerph18157771

**Published:** 2021-07-22

**Authors:** Abdulazeem S. Alotaibi, Boukhemis Boukelia

**Affiliations:** 1 Department of Education and Movement Sciences, College of Education, Qassim University. P.O. Box 6688, Qassim 51452, Saudi Arabia; atiebya@qu.edu.sa; 2 Britannia Sport and Exercise Science Academy, 6/7 Duff Street Lane, Edinburgh EH11 2HS, UK

**Keywords:** COVID-19, physical activity, physical inactivity, BDI, KSA, depression

## Abstract

The outbreak of COVID-19 and the changes to normal societal function and in particular quarantine has increased mental distress in many nations. A survey of 22,112 COVID-19-negative quarantined participants in the Kingdom of Saudi Arabia (age: 18–40 years, 42.6%; 40–60 years, 53.3%; over 60 years, 4.1%; mass, 78.9 ± 14.8 kg; stature, 167 ± 8.7 cm) were assessed for depressive symptoms using the online Beck Depression Inventory self-report questionnaire. The relationship between pre-quarantine physical activity and mental health and wellbeing during lockdown has been investigated. A significant difference in body mass index (BMI) between active and inactive participants (*p* = 0.03) was observed; with females also recording a 3% higher BMI than males. All participants showed a decrease in mental health compared to pre-quarantine. However, pre-quarantine inactivity was found to result in a greater negative impact on mental health and well-being than those active pre-quarantine (*p* < 0.01). The sedentary population had a 4-fold greater incidence of mild-depression than the active population. This suggests that activity level plays an important role in shielding people from anxiety and stress, whilst it builds mental strength in individuals that can be called upon in trying and difficult situations. Nevertheless, pre-quarantine activity levels did not lead to any significant change in levels of extreme depression in the sample population.

## 1. Introduction:

Most countries affected by the SARS-CoV-2 (COVID-19) have initiated a variety of social restrictions, with quarantine used to isolate individuals to prevent the spread of infection. However, despite these drastic measures being used for the greater public good, quarantine itself can manifest a range of psychological problems and diminish human well-being due to separation from family, friends and loved ones, and prolonged periods of limited to no social interaction [1,2].

Several studies have reported a high prevalence of psychological distress symptoms including depression and anxiety among quarantined people [1,3,4,5,6]. Symptoms of acute stress disorder have been shown in staff from a Taiwanese hospital after 9 days quarantine due to the SARS pandemic 2003 outbreak [7]. In the same study, quarantined staff were significantly more likely to report anxiety when dealing with febrile patients, exhaustion, detachment from others, irritability, and insomnia.

In support of these findings, during the Australian epidemic of highly infectious equine influenza in 2007, 2760 horse owners were quarantined for four weeks, 34% of whom reported elevated psychological distress compared to 12% in the general population [8]. Furthermore, enforced isolation during the Middle East Respiratory Syndrome epidemic resulted in negative affects to mental health with 17% reporting a feeling of rage and 7% displaying symptoms of anxiety [9]. Hall et al. [10] found that many individuals reported a high degree of anxiety and fear of dying or becoming ill during the Ebola epidemic, which may have caused a mental breakdown.

Recent studies assessing the impact of the initial outbreak of SARS-CoV-2 within the USA found that 45% of respondents reported increased stress and worry as a result of quarantine [6]. Likewise, Wang et al. [5] found that 53.8% of respondents reported moderate to extreme depressive symptoms as a result of quarantine measures.

The dissatisfaction of not being able to engage in normal day-to-day activities, the boredom induced by containment, fear of dying or becoming ill and a lack of normal routine have been correlated with an increase in the level of stress among the quarantined population [2,11]. Furthermore, it was also a source of dissatisfaction to have insufficient daily basic supplies, [12] primarily food, during the quarantine period such as in the study by Cava et al. [2] where participants reported obtaining their purchases late or not at all, such as thermometers and masks. Additional, frustration with insufficient supplies appeared to be associated with anxiety and indignation many months after quarantine [9].

Nevertheless, physical activity has been shown to be as effective as antidepressants in decreasing stress, enhancing self-esteem, and improving mood [13,14,15,16]. Despite the known benefits of physical activity, three quarters of the adult population in Saudi Arabia are classified as inactive [17]. However, the closure of public activity spaces, social distancing restrictions and collective physical activity was prohibited due to COVID-19 restrictions; this in turn will likely increase the rates of physical inactivity in this nation [18]. In contrast many nations have embraced daily physical activity among the quarantined population as a beneficial way to self-manage mental health stress and may attenuate mental health and wellbeing decline resulting from COVID-19 [19].

The impact of separation from loved ones, loss of freedom, uncertainty over disease status, boredom and physical inactivity are key factors which dramatically increase stress and anxiety levels among a quarantined population. The present study aimed to examine the effects of quarantine on mental health and well-being in the population of Saudi Arabia and the extent to which pre-quarantine physical activity can protect against the decline in mental health and wellbeing during lockdown.

## 2. Method

### 2.1. Participants

In total, 9845 female and 12,208 male participants aged between 18 and 70 years old from the Kingdom of Saudi Arabia took part in the study. Participants are divided into 3 age groups 18–40 years old, 41–60 years old and over 60 years old. Participants were recruited on a voluntary basis by canvassing on social media, local radio stations and through university mailing lists. None of the participants reported as having tested positive for COVID-19, either before or during the study period.

### 2.2. Procedures

This study is a qualitative analysis of the Beck Depression Inventory (BDI) Scale for a quarantined population. Physical activity levels pre-quarantine and during quarantine are correlated with depression levels to determine any relationships. Data collection took place from 1 March 2020 to the 30th of April 2020 in the Kingdom of Saudi Arabia using an online questionnaire platform that also collected demographic information.

At the onset of data collection, coronavirus restrictions and school closures in the Kingdom of Saudi Arabia had been in place for 8 weeks and remained unchanged during data collection.

### 2.3. Questionnaire

A self-report questionnaire, the online Beck Depression Inventory (BDI) second edition [20], was used in this study to assess depressive symptoms in COVID-19-negative participants based in the Kingdom of Saudi Arabia. A 21 multiple-choice self-rated scale evaluates key symptoms of depression including work difficulty, crying, guilt, indecisiveness, loss of libido, self-dislike, pessimism, mood, sense of failure, punishment, insomnia, self-accusation, fatigability, loss of appetite, weight loss, suicidal ideas, self-dissatisfaction, body image change, social withdrawal, somatic preoccupation and irritability. Individual scale items are scored on a 4-point continuum (0 = least, 3 = most), with a total summed score range of 0–63. Higher scores indicate greater depressive severity.

### 2.4. Physical Activity

Physical activity was measured using a series of direct questions in which participants selected which activity level represented their physical activity pre-quarantine and during the quarantine period [21]. The physical activity levels are defined as active or inactive based on the WHO criterion for activity [22]. The WHO recommends that all adults should undertake 150–300 min of moderate-intensity exercise per week, or 75–150 min of vigorous-intensity physical activity per week, or some equivalent combination of moderate-intensity and vigorous-intensity aerobic physical activity, per week. Active participants are described as those who undertook a minimum of 150 min of physical activity a week or engaged in vigorous activity of greater than 75 min per week. Whereas inactive participants are those that exercise less than 150 min of moderate-intensity activity per week (or less than 75 min of vigorous-intensity exercise per week) or not at all. Active participants were asked to state how many times a week that they engaged in physical activity, and the duration(s) and quality (moderate-vigorous intensity) of the exercise performed.

### 2.5. Assessing Mental Health and Wellbeing

Mental health was assessed using the online Beck Depression Inventory (BDI). BDI scores are summed to create a total score, with lower scores indicating lower levels of depression. Physical activity levels are assessed using the WHO criterion with correlations between BDI scores and physical activity levels used to assess mental health and well-being. BMI has also been used as a wellbeing marker alongside the physical activity levels.

### 2.6. Statistical Analysis

Prior to statistical analysis, all data were checked for normality. Repeated measure T tests were used to determine significance difference in BMI for the female and male study populations (SPSS26 Statistical Software, IBM. London, UK). The effects of physical activity levels on BMI, in gender and BDI were determined using a two-way repeated-measures ANOVA. Statistical significance was accepted at *p* < 0.05. Results are represented as mean values ± standard deviation (SD). Cohen’s d was used to assess the size of the difference between two related sample.

## 3. Results

Table 1 showing general participant information including gender, educational level, age, and BMI. There was a significant difference in score in female BMI and male condition t (13,275) = 4.75, *p* = 0.01 (*) with females showing 3% higher value of BMI than male. Higher BMI has a significant negative effect on mental well-being in this study (F_78, 21958_ = 12.08, *p* < 0.001, partial η^2^ = 0.45), in addition, this significant difference is evident in the male and female populations (F_2,22051_ = 20.05, *p* < 0.001, partial η^2^ = 0.02).

**Figure 1:**
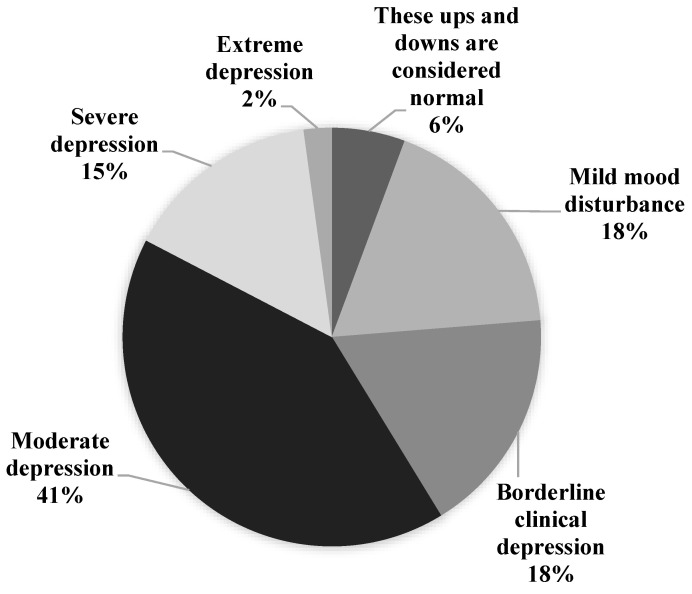
Beck’s Depression Inventory scores for the total population showing the spectrum of depression experienced during quarantine.

From the total sample population, 41.4% showed moderate depression (45% female vs. 55% male), 15.2% (49% female vs. 51% male) showed severe depression, and 2.2% (48 female vs. 52 male) showed symptoms of extreme depression (Figure 1). There was a significant difference in gender for overall mental wellbeing (F_2,22051_ = 20.02, *p* < 0.001, partial η^2^ = 0.02). Females exhibited moderate depression at 42% vs. 41% for males, borderline clinical depression in females was 17.5% vs. 17.8% for males, and mild mood disturbance in females was exhibited by 16.7% vs. 18.6% for males. Both severe and extreme depression were higher in females than males, at 16.3% vs. 14.5% and 2.3% vs. 2.1%, respectively.

Inactivity pre-quarantine comprised 77.08% of the overall participants. A further 9.89% exercised less than three-times a week, with only 13.02% exercising more than three-times a week, hence fulfilling the WHO activity guideline levels (Table 2). There was a significant positive relationship between inactivity and BMI (t (22,052) = 3.97, *p* = 0.01), with the female population recording a significantly higher BMI than the male population (t (22,051) = 7.89, *p* = 0.03).

There was a significant difference in mental wellbeing observed during quarantine between those defined as active compared to inactive before the quarantine period (F_1,22052_ = 4.08, *p* < 0.001, partial η^2^ = 0.2). The pre-quarantine physically active population showed 8% moderate depression symptoms, 2% showed severe depression symptoms and 0.4% showed extreme depression symptoms (Table 3). Higher pre-quarantine physical activity had a significant positive effect on mental health during quarantine compared to the active population during the quarantine (47% vs. 26%).

## 4. Discussion

This study focused on the effect of pre-quarantine physical activity on anxiety and depressive symptoms during the COVID-19 lockdown in the Kingdom of Saudi Arabia and provides some evidence that pre-quarantine physical activity can be considered as a strategy to improve health and manage mental well-being during this pandemic or similar future events.

During the quarantine period, this study found that 41.4% exhibited at least moderate depression with 15.2% exhibiting severe or extreme depression from the total sample population. Females exhibited more severe, extreme, and moderate depression compared to the males that exhibited mild and borderline depression. These findings are supported by Vizard et al. [23] that found one in five adults were likely to be experiencing some form of depression during the lockdown conditions because of the COVID-19 pandemic. Whereas, 1 in 10 experienced some form of depression pre-pandemic [23]. Importantly, our study demonstrates that a physically active population pre-quarantine shows a four-fold lower incidence of moderate depression compared to a sedentary population. This finding supports the hypothesis that individuals that are physically active have a higher degree of mental strength and are therefore more able to endure trying and difficult circumstances [18].

There was an increase in inactivity by 21% during the quarantine period compared to pre-quarantine period in Saudi Arabia. This contrasts with research findings from other nations, such as Italy and Spain, that have shown increased levels of physical activity during the lockdown periods [24,25]. It may be that this rise in physical activity among the population of some nations are due in part to the strategy of these nations by allowing and recommending daily exercise, preferably outdoors (including permitting short travel distances but excluding indoor activities) with the purpose of engaging in physical activity.

It is well known that exercise reduces stress and anxiety, however, this research has shown that participants that maintained physical activity during quarantine still expressed symptoms of depression, with 9.15% having moderate symptoms of depression, 2.5% having serious symptoms of depression, and 0.3% having extreme symptoms of depression. This phenomenon may be due to separation from family, friends and loved ones and prolonged periods of limited to no social interaction. Undeniably, a clear association has been documented between mental health and quarantine period in the literature [11,26,27]. The length of quarantine, severity of restrictions and the fear of infection are listed as the main stressors among the population [1].

To the best of our knowledge, most of our physically active participants (exercising more than three times a week), were engaged in indoor physical activity only, due to a lockdown “stay at home” order. It is suggested that only exercising indoors may have a lesser positive affect on mental health and therefore has not eased the depressive symptoms compared with exercising outdoors that allows people to connect with nature [28]. This is supported by the research of Lawton et al. [28] that observed indoor physical activity was associated with high levels of anxiety and stress compared to exercising outdoors. Furthermore, that outdoor physical activity and nature-related emotions improve psychological wellbeing and mental status [28]. In conjunction, the disruption of daily routines and the boredom induced by the quarantine measures adds another dimension to this phenomenon. During the quarantine period, at least four out of ten people showed symptoms of depression from the total sampled population (active and inactive). Nevertheless, physical exercise prior to quarantine played a significant role in protecting people from stress and anxiety during this time.

It is important to note that only 12.8% of the participant population qualified as physically active that met the WHO criteria for physical activity. The average BMI is 28.24 ± 5.24 for males and 29.02 ± 6.95 for females; suggesting that the sampled population is at an unhealthy weight. This random population sample has demonstrated a concerningly low percentage of active population in the Kingdom of Saudi Arabia. Society should be alerted to the need for an emergency plan to promote exercise for a myriad of health benefits, both for physical and mental well-being, including a reduction of the likelihood of developing mild anxiety and depression under stressful conditions such as quarantine.

This study supports the existing literature reporting that anxiety and stress is lower in the pre-quarantine active population compared to a sedentary population under quarantine. These active individuals are better equipped to deal with the mental challenges faced during quarantine and a global pandemic. Therefore, promotion of exercise within the Kingdom of Saudi Arabia will improve the physical and mental health and well-being of the population. A national strategy of increased physical activity is recommended. Moreover, considerations for future quarantine rules should examine the possibility of outdoor exercise, that was promoted in other countries that may improve mental health through a connection to nature or by simply creating a change of scenery.

The study considered qualitative analysis only and could be improved or made more robust by utilizing biochemical tests, such as the measurement of an instant cortisol level and/or chronic stress levels, by the assessment of cortisol concentrations in the hair in concert with other qualitative methods. A limitation of this study that should be improved upon by future research is to increase the number of physically active participants in the sample population pre-COVID-19, to render the results as more robust. An additional limitation is to investigate if gender, age, ethnicity or other factors increase the likelihood of depressive symptoms during quarantine regardless of physical activity levels.

## Figures and Tables

**Table 1:** General participant information.

Age Group	%	Gender		Marital Status		
18–40	42.6%	Male	Female	Married	Single	Divorced
41–60	53.3%	55.5%	44.5%	61.3%	28.9%	9.8%
Over 60	4.1%					
Educational Level	N/certificate	High school		Undergraduate	Postgraduate	
	4.7%	16.7%		58.1%	20.5%	
BMI	Male			Female		
	28.25 ± 5.24 *			29.02 ± 6.95 *		

* denotes a significant statistical difference is observed.

**Table 2:** The level of physical activity pre-COVID-19 quarantine vs. level of activity during COVID-19.

Group	Inactivity		>3 Time a Week		Less Than 3 Times a Week	
Pre-COVID-19 quarantine activity level	17,045	77.08%	2880	13.02%	2188	9.89%
During COVID-19 quarantine activity level	17,694	80.01%	2250	10.17%	2169	9.80%
Δ	649	−2.93%	−630	−2.85	−19	0.09%

**Table 3:** Pre-quarantine and during quarantine physical active people BDI score.

Levels of Depression	Pre-Quarantine Physical Active		During Quarantine Physical Active	
Total physically active	5068	100%	4419	100%
Moderate depression	406	8%	643	14.6%
Severe depression	110	2%	144	3.25%
Extreme depression	12	0.4%	43	1%
Borderline clinical depression	989	19.5%	1076	24.5%
Mild mood disturbance	1177	23%	1359	30.7%
These ups and downs are considered normal	2374	47%	1154	26%

## Data Availability

Restrictions apply to the availability of these data. Data was obtained from Qassim University Saudi Arabia and are available from the authors with the permission of Al Qassim University.
